# The intake of maqui (*Aristotelia chilensis*) berry extract normalizes H_2_O_2_ and IL-6 concentrations in exhaled breath condensate from healthy smokers - an explorative study

**DOI:** 10.1186/s12937-015-0008-1

**Published:** 2015-03-19

**Authors:** Daniela Vergara, Daniela Ávila, Elizabeth Escobar, Catalina Carrasco-Pozo, Andrés Sánchez, Martin Gotteland

**Affiliations:** Department of Nutrition, Faculty of Medicine, University of Chile, Santiago, Independencia 1027, Independencia, Santiago Chile

**Keywords:** Smokers, H2O2, IL-6, Exhaled breath condensate, *Aristotelia chilensis*, Anthocyanins, Respiratory diseases, Oxidative stress, Inflammation

## Abstract

**Background:**

Respiratory diseases are associated with pulmonary oxidative stress and inflammatory processes. Though studies in animal models suggest that dietary polyphenols improve lung injury, no intervention studies were carried out in humans. The aim of this study was to determine whether the intake of an anthocyanin-rich maqui extract improved H_2_O_2_ and IL-6 concentrations in exhaled breath condensates (EBCs) from asymptomatic smokers.

**Findings:**

15 asymptomatic smokers with mild cigarette smoking (3 pack-year [2.4 - 7.7]) (mean [CI_95%_]) were recruited in this exploratory longitudinal study. They ingested 2 g of maqui extract (polyphenol content = 5.18 ± 2.00 g GAE/100 g; FRAP value = 27.1 ± 2.0 mmol Fe^++^/100 g), twice daily for two weeks. EBCs were collected before and after treatment and the changes in H_2_O_2_ and IL-6 concentrations were determined by fluorimetry and Elisa, respectively. The EBC contents of H_2_O_2_ and IL-6 H_2_O_2_ before and after treatment in smokers were also compared with those determined in single EBC samples from 8 healthy non-smokers subjects. At baseline, the H_2_O_2_ concentrations were higher and those of IL-6 lower in the smokers than in the non-smokers. Maqui extract significantly decreased H_2_O_2_ (p < 0.0002) and increased IL-6 (p < 0.004) in the EBC from smokers. The EBC concentrations of H_2_O_2_ and IL-6 after maqui administration did not differ between smokers and non-smokers.

**Conclusions:**

Maqui extract normalizes IL-6 and H_2_O_2_ concentrations in EBC from humans with mild smoking habits. If confirmed, these results suggest that dietary polyphenols might be considered as an interesting alternative for the dietary management of respiratory disorders.

## Findings

Respiratory diseases including pneumonia, tuberculosis, asthma, cystic fibrosis, emphysema and chronic obstructive pulmonary disease are associated with oxidative and inflammatory processes generated by microorganisms, air pollutants or tobacco smoke. Epidemiological studies suggest that a high intake of fruits and vegetables exerts health-promoting effects on lung injury and function [1], probably due to the abundance of vitamins and polyphenols with antioxidant, anti-inflammatory or mitochondrial regulatory properties in these foodstuffs [2]. This is also supported by the fact that deficiencies in antioxidant vitamins are frequently described in patients suffering respiratory diseases while intervention studies indicate that vitamin supplementation might improve their health state [3,4]. On the other hand, flavonoid-rich diets are negatively associated with chronic cough and it has been shown that apple intake, as well as that of red wine, decreases the risk of asthma [5,6]. Though experimental studies indicate that polyphenol administration improves lung injury in animal models, no intervention studies about this matter were carried out in humans.

Maqui (*Aristotelia chilensis*) is an edible berry endemic from central and southern Chile. It exhibits one of the highest polyphenol content and antioxidant activity of all fruits including the most consumed berries, being particularly rich in the anthocyanins delphinidin and cyanidin [7-9]. Maqui has been traditionally used in the Chilean folk medicine for its antidiarrheic, anti-inflammatory and antipyretic properties; extracts of this berry are actually commercialized as nutraceuticals, mainly based on their high antioxidant activity. More recently, maqui polyphenols were shown to protect mice against ischemia–reperfusion-induced heart damage [10], to inhibit adipogenesis and inflammation *in vitro* [11] and to prevent LDL oxidation [12]. Maqui also improved hyperglycemia and insulin resistance in obese mice fed a high fat diet, probably through the modulation of glucose metabolism in the skeletal muscle and liver [13].

Based on these findings, we hypothesize that the intake of maqui extract improves the concentrations of hydrogen peroxide (H_2_O_2_) and IL-6 in the exhaled breath condensate (EBC) from asymptomatic smokers. We selected H_2_O_2_ and IL-6 as biological markers due to the fact that these have been widely used in many studies to evaluate the oxidative and immune changes occurring in smoker subjects and patient with lung diseases. On the other hand, the rational to carry out the study in asymptomatic smokers rather than in symptomatic heavy smokers is that it was more probable to detect a protective effect of the maqui extract in subjects with mild alteration of their pulmonary oxidative/inflammatory status.

### Study design

The study was approved by the Ethics Committee of the Faculty of Medicine, University of Chile; each volunteer signed an informed consent before its inclusion in the protocol. Exclusion criteria included pregnancy, acute or chronic digestive or respiratory pathologies, type-2 diabetes, autoimmune or allergic diseases, acute or chronic intake of drugs or vitamin supplements. This was an exploratory, open and uncontrolled study carried out in 15 asymptomatic smokers who exhibited a low intake of fruits and vegetables (<200 g/d, i.e. lower than half of the WHO recommendations) and a moderate cigarette consumption (mean [CI_95%_]) (smoking habits: 8 cigarettes/day [7 - 15]; smoking history: 6 y [5 - 13 y]; number of pack-year: 3 [2.4 - 7.7]). The volunteers had to ingest 2 g of a maqui extract (a gift of Nativ for Life, Santiago, Chile) twice daily for two weeks. The extract displayed a total polyphenol content of 5.18 ± 2.00 g of Gallic Acid Equivalents (GAE)/100 g and a FRAP (Ferric Reducing Antioxidant Power) value of 27.1 ± 2.0 mmol Fe^++^/100 g, as determined in our laboratory [14,15]. The nutritional composition of the extract per 2 g-serving was: energy: 5 kcal; protein: 0.1 g; fat: 0.2 g; carbohydrate: 0.6 g; total fiber: 1 g and sodium: 0.4 mg. During this two-week period, digestive symptoms and stool frequency and consistency were daily registered by the volunteers using an *ad hoc* form and the Bristol Scale for stool evaluation. The subjects continued smoking cigarettes during the treatment period. The samples of EBC were collected before and after the administration of the maqui extract in each of the participants. For comparison, a single sample of EBC was also obtained from each one of 8 non-smokers healthy subjects; these subjects did not receive any maqui extract.

### Determination of H_2_O_2_ and IL-6 in exhaled breath condensate

The collection of EBC samples was carried out as described elsewhere [16]. Subjects had to breathe for 10 min at a normal frequency and tidal volume in an EcoScreen apparatus (Jaeger, Wurzburg, Germany) equipped with a mouthpiece and a two-way non re-breathing valve also serving as a saliva trap. The condensates (~ 1 mL) were maintained frozen until use. The concentrations of H_2_O_2_ in EBC was measured in a 96-wells microplate by using Amplex® Red reagent (Life Technologies, Carlsbad, CA, USA). Amplex Red, in the presence of peroxidase, reacts with H_2_O_2_ in a 1:1 stoichiometry to produce resorufin, a red fluorescent compound with an absorption/emission maxima of 570/585 nm. Fluorescence was determined in a Synergy HT™ multi-detection Microplate reader (BioTek Instruments, USA); the limit of detection of the method was 4 nM (i.e. 200 fmoles/well). IL-6 concentrations were determined using a commercial human IL-6 Elisa kit (Pierce, USA, respectively); the sensibility of the assay was <1 pg/ml. The coefficients of variation intra- and inter-assay for these determinations was <15%.

### Statistical methods

Results were presented as means [CI_95%_]. Normality of the variables was determined by the Shapiro-Wilk test. Changes in H_2_O_2_ and IL-6 values after treatment in the smokers were analyzed by paired Student's t-test. As the variances of both groups were not homogenous, H_2_O_2_ and IL-6 values between groups were compared by using the non-parametric Mann-Whitney U test.

## Results

**Figure 1:**
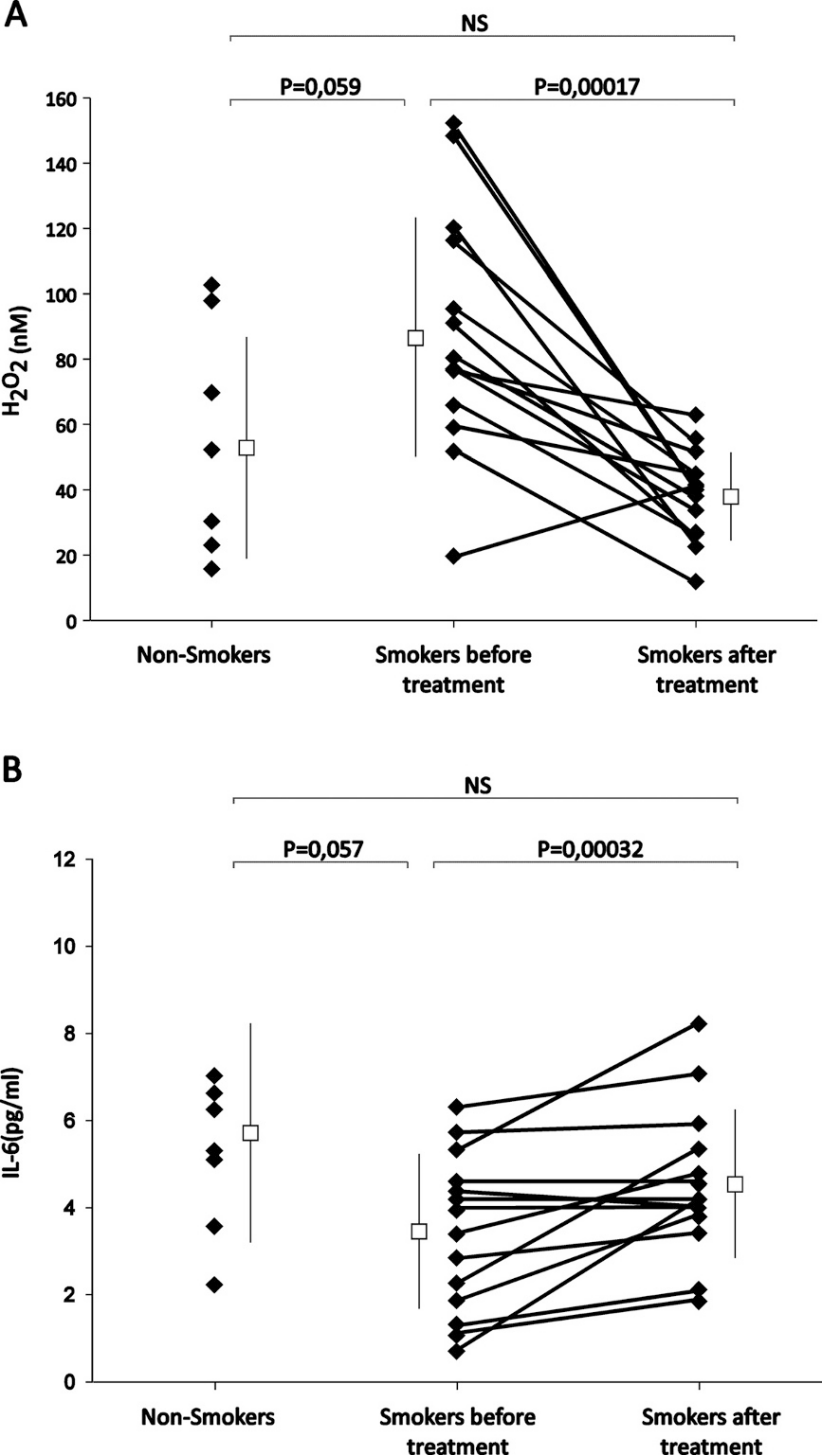
**Concentrations of H**_**2**_**O**_**2**_**(A) and IL-6(B) in exhaled breath condensates from non-smoker controls subjects and smokers before and after the two-week administration of maqui extract.** The squares and vertical bars indicate the means and CI_95%_.

The characteristics of both groups of subjects are described in Table 1. Before initiating maqui administration, the EBC concentration of IL-6 in smokers tended to be lower than those detected in the non-smokers (3.64 pg/ml [2.59 – 4.69] *vs*. 5.71 pg/ml [3.59 – 7.82], respectively; p = 0.057) and that of H_2_O_2_ higher (88.6 nM [67.6 – 109.5] *vs*. 52.5 nM [24.3 – 80.7], respectively; p = 0.059) (Figure 1). Although various studies reported higher pulmonary concentrations of IL-6 in smokers, they are generally carried out in older subjects, with a more elevated consumption of cigarettes and a longer smoking history, than the subjects who participated in our study [14]. Our results confirm those from McCrea et al. [17] who described that the IL-6 concentrations in bronchoalveolar lavage fluid (BALF) are lower in healthy smokers than in nonsmokers, and that BALF macrophages isolated from these smokers released less IL-6 when stimulated with LPS. Similar results have been reported in a number of studies that described a lower release of bioactive IL-6 and other pro-inflammatory cytokines from alveolar macrophages in smokers than in nonsmokers [18-22]. This immune alteration might explain the delayed rate of bacterial clearance in mice exposed to cigarette smoke and posteriorly infected with *P. aeruginosa* [23] and the higher susceptibility to pulmonary infections in smokers. Various components of tobacco including acrolein, nicotine, tar, hydroquinone and catechol have been shown to inhibit the immune response [24]. In addition, it has also been suggested that exposure of alveolar macrophages to tobacco smoke results in a hyporesponsive state similar to endotoxin tolerance, due to the inhibition of the TLR2/4-induced expression of pro-inflammatory cytokines and the impaired activation of IRAK-1, p38, and NF-ķB [25]. 4-(Methylnitrosamino)-1-(3-pyridyl)-1-butanone, a component of tobacco smoke has also been shown to decrease IL-6 from bronchial and alveolar epithelial cells [26].

**Table 1:** **Characteristics of the control (non-smoker) and smoker groups**

	Non-smokers (n = 8)	Smokers (n = 15)
Female (%)	62.5	40
Age (y)	22.0 ± 1.1	26.4 ± 8.6
BMI (kg/m2)	23.1 ± 3.4	25.0 ± 1.8

Our results also confirm previous studies indicating higher H_2_O_2_ levels in EBC from smokers [27]. The smoker subjects consumed the maqui extract daily for two weeks. The treatment was well tolerated by the subjects and no side-effect, including digestive symptoms, were reported during the study. At the end of the treatment period, a significant decrease (by 57.3%) of H_2_O_2_ concentrations (37.8 nM [30.0 – 45.8]; p = 0.0002) and a significant increase of IL-6 concentrations (by 30.8%) (4.76 pg/ml [3.72 - 5.79]; p = 0.0032) were observed. Such changes in H_2_O_2_ and IL-6 were detected in 92.8% and 80% of the smokers, respectively; they were inversely correlated (R = 0.59; p = 0.027). At the end of the period of maqui administration, the EBC concentrations of these molecules in the smoker subjects did not differ from these detected in the non-smokers. Some studies have described the ability of polyphenols to act as immunostimulant and more specifically to increase IL-6 expression. Sage phenolic compounds, for example, increased IL-6 and TNF release by the macrophage-like cells RAW264.7 infected by Leishmania [28] while grape seed extract increased IL-6 expression and secretion in astrocytes, improving their resistance against H_2_O_2_-induced oxidative damage [29]. To our knowledge, our results show for the first time that the intake of a polyphenol-rich extract exerts beneficial effects in the respiratory tract of humans, normalizing the concentrations of IL-6 and H_2_O_2_ EBC in asymptomatic smokers with moderate cigarette smoking. Such protective effects have been described in animal models of respiratory diseases with different dietary polyphenols. For example, resveratrol was shown to improve lung injury induced by staphylococcal enterotoxin-B in mice [30] while the administration of a walnut extract restored the levels of glutathione reductase and catalase and reduced the xanthine oxidase activity in lung tissues of rats treated with cigarette smoke extract [31]. Another widely used dietary polyphenol, curcumin, was shown to attenuate the pulmonary inflammation and emphysema induced by intratracheal porcine pancreatic elastase or cigarette smoke in mice [32]. Polyphenols from red wine and red fruits were also shown to cause bronchodilation and to suppress airway inflammation in an animal model of asthma induced by ovalbumin sensitization [33]. Berry anthocyanins have been shown to be absorbed through the intestinal mucosa in *in vitro* system (Caco-2 cells) and in human volunteers [34,35], therefore making possible that these molecules may reach the respiratory tract and exert their antioxidant and anti-inflammatory activities. This was recently supported by the observations from Aquil et al. who detected the presence of different anthocyanins in the lung tissue from blueberry-fed mice [36].

## Conclusions

These preliminary results suggest that the intake of a dietary polyphenol-rich maqui extract increases the EBC concentrations of IL-6 and decrease those of H_2_O_2_ in humans with mild smoking habits. Such changes could be beneficial for the subject as it could improve their resistance to respiratory infections while lowering oxidative stress in their lungs. These results support the realization of randomized, double-blind, placebo-controlled clinical trials in higher numbers of subjects, smokers or patients with respiratory diseases, to confirm such effect. If so, dietary polyphenols might be considered as an interesting alternative for the dietary management of these pathologies.

## Abbreviations

CI_95%_: Confidence Interval 95%
EBC: Exhaled breath condensate
H_2_O_2_: Hydrogen peroxide
IL-6: Interleukin-6
